# WormBase 2014: new views of curated biology

**DOI:** 10.1093/nar/gkt1063

**Published:** 2013-11-03

**Authors:** Todd W. Harris, Joachim Baran, Tamberlyn Bieri, Abigail Cabunoc, Juancarlos Chan, Wen J. Chen, Paul Davis, James Done, Christian Grove, Kevin Howe, Ranjana Kishore, Raymond Lee, Yuling Li, Hans-Michael Muller, Cecilia Nakamura, Philip Ozersky, Michael Paulini, Daniela Raciti, Gary Schindelman, Mary Ann Tuli, Kimberly Van Auken, Daniel Wang, Xiaodong Wang, Gary Williams, J. D. Wong, Karen Yook, Tim Schedl, Jonathan Hodgkin, Matthew Berriman, Paul Kersey, John Spieth, Lincoln Stein, Paul W. Sternberg

**Affiliations:** ^1^Informatics and Bio-computing Platform, Ontario Institute for Cancer Research, Toronto, ON M5G0A3, Canada, ^2^Genome Sequencing Center, Washington University, School of Medicine, St Louis, MO 63108, USA, ^3^Division of Biology and Biological Engineering 156-29, California Institute of Technology, Pasadena, CA 91125, USA, ^4^European Molecular Biology Laboratory, European Bioinformatics Institute, Wellcome Trust Genome Campus, Hinxton, Cambridge CB10 1SD, UK, ^5^Department of Genetics Campus, Washington University School of Medicine, St. Louis, MO 63110, USA, ^6^Genetics Unit, Department of Biochemistry, University of Oxford, Oxford OX1 3QU, UK, ^7^Wellcome Trust Sanger Institute, Wellcome Trust Genome Campus, Hinxton, Cambridge CB10 1SA, UK and ^8^Howard Hughes Medical Institute, California Institute of Technology, Pasadena, CA 91125, USA

## Abstract

WormBase (http://www.wormbase.org/) is a highly curated resource dedicated to supporting research using the model organism *Caenorhabditis elegans*. With an electronic history predating the World Wide Web, WormBase contains information ranging from the sequence and phenotype of individual alleles to genome-wide studies generated using next-generation sequencing technologies. In recent years, we have expanded the contents to include data on additional nematodes of agricultural and medical significance, bringing the knowledge of *C. elegans* to bear on these systems and providing support for underserved research communities. Manual curation of the primary literature remains a central focus of the WormBase project, providing users with reliable, up-to-date and highly cross-linked information. In this update, we describe efforts to organize the original atomized and highly contextualized curated data into integrated syntheses of discrete biological topics. Next, we discuss our experiences coping with the vast increase in available genome sequences made possible through next-generation sequencing platforms. Finally, we describe some of the features and tools of the new WormBase Web site that help users better find and explore data of interest.

## INTRODUCTION

*Caenorhabditis elegans* is a free-living soil nematode found throughout the world. Its small size (1 mm), rapid generation time (∼3 days), simple nervous system and invariant developmental program have made it a well-known system for studying a broad array of biological problems [(1,2); http://www.wormbook.org]. WormBase aims to facilitate and accelerate research using *C. elegans* through a process of deliberate and detailed curation of the primary literature.

When launched, WormBase expanded prior community-driven curation to touch on virtually every aspect of classical and modern experimental biology, including next-generation sequence and high-throughput data. As these efforts continue, we are expanding our focus to create synthesized views of the scientific knowledge contained in WormBase. These ‘biological topics’ represent large and complex problems not readily described through gene-by-gene curation and not always represented in the primary literature.

Next-generation sequencing technology has had a tremendous impact on the direction of curatorial efforts at WormBase. These include an exploration of natural variation in *C. elegans* and a constant stream of whole-genome sequences and preliminary annotation from related species. We balance inclusion of these data sets based on potential value to our user community and resources required to adequately import data into WormBase.

To support increased demand for WormBase, changing user expectations and constantly growing data requirements, we have redesigned the WormBase Web site from the ground up. Launched in March 2012, the new site offers users the option to customize the content and arrangement of pages to suit individual needs. WormMine, a new data mining tool using the Intermine data mining platform, was launched offering new options for querying and interacting with data at WormBase.

## BIOLOGICAL TOPICS CURATION

For 13 years, WormBase curators have been collecting data of various types pertaining to the biology of *C*. *elegans* and related nematodes. These data types have included gene models, allelic variations, mutant phenotypes, anatomy function, expression patterns, gene interactions (physical, genetic and regulatory) and, more recently, human disease relevance. Although these various data types have existed predominantly in isolation, WormBase is now aiming to synthesize integrated views of these data in the form of ‘Biological Topics’, big-picture perspectives that draw together all data relevant to a biological topic of interest. *C. elegans* has proven to be a tremendously useful model organism for the study of many topics, including cell death and differentiation, embryogenesis, organ development and aging. Much of this important research has been summarized in the online ‘WormBook’ (http://www.wormbook.org/), a collection of review articles written by the nematode research community. The content of WormBook has inspired the creation of the first generation of WormBase Biological Topics, including behavioral topics such as locomotion, foraging and male mating; cellular topics such as cell fusion, cell migration and cell death; and signaling pathway topics such as RTK/Ras/MAPK, EFG and Notch.

Researchers who come to the WormBase Web site with a particular goal of understanding how nematode research has informed a particular field of study are now able to explore WormBase data from a perspective that most closely pertains to their field of inquiry. Whether researching a human disease or studying a molecular mechanism, users can search for their topic and review the relevant WormBase data in an intuitive manner. Each WormBase Biological Topic has a dedicated web page for displaying all relevant WormBase entities. In addition to a curator-generated text summary, the page lists relevant genes, phenotypes, anatomy terms, life stages, gene expression clusters, interactions, molecules (small molecules, chemicals, drugs), Gene Ontology (GO) terms, human diseases and publications. The connections of WormBase entities, such as genes or phenotypes, to a particular Biological Topic are curator confirmed, ensuring high-quality annotations. A cytoscape-based interaction viewer allows users to see all genetic, physical and regulatory interactions that affect the topic of interest. These interaction network views can be filtered to allow closer inspection of certain types of interactions (regulatory versus genetic) or associated phenotypes (for genetic interactions).

In addition, the Biological Topic page may include one or more depictions of relevant pathways, whether they be molecular signaling pathways, or more large-scale cell–cell interaction pathways, for example. WormBase works with WikiPathways (http://www.wikipathways.org) to generate pathway diagrams for *C. elegans* and related nematodes to be displayed on WormBase Biological Topic pages. The WikiPathways approach provides the benefit that many WormBase curators or experts in the field may simultaneously create, develop and maintain a common pathway, or depict alternate pathways, of a Biological Topic. WormBase curators will review nematode pathways on WikiPathways and provide official approval to pathways that meet certain quality criteria, such as proper citations and evidence. Once approved, these pathways will be incorporated into WormBase Biological Topic pages. WikiPathways has a specific WormBase ‘Portal’ page (http://www.wikipathways.org/index.php/Portal:WormBase) that directly links users to nematode pathways of interest. WikiPathways currently houses >50 *C. elegans* pathways, nine of which are WormBase approved.

In an effort to coordinate curation effort and most effectively synthesize the Biological Topic pages described earlier, the WormBase literature curation pipeline has undergone some changes. Previously, curators went through publications paper by paper to extract specific data types. Now, we concentrate on one Biological Topic at a time, extracting all relevant data in the literature. From this collection of information, we can then generate the most comprehensive and up-to-date view of the topic.

## GENOMES AND SPECIES

### The *C. elegans* reference genome and sequence annotation

Careful manual curation of the *C. elegans* reference genome sequence and annotation continues to be a key activity for WormBase. We have recently released a new version of the reference genome (WBcel235) that includes 1402 corrections, drawn and reviewed from a number of independent projects that have re-sequenced the Bristol N2 reference strain (3–5). Active refinement of the canonical gold-standard set of structures for protein-coding genes, non-coding RNA genes, pseudogenes, transposons and operons also continues, using experimental data drawn from a wide variety of sources and tools developed within project (6,7).

### *C. elegans* natural variation

The past 2 years have seen rapid growth in volume and diversity of nematode genomic variation data, in large part due to various projects engaged in whole-genome sequencing of hundreds of *C. elegans* wild-isolate strains (8,9). We have responded to this challenge by making changes to the way in which we curate, store and display variation data. One significant change has been to clearly distinguish between naturally occurring polymorphisms and laboratory-induced mutations, at both the database and display levels. We have also consolidated redundant data from independent wild-isolate sequencing projects, creating reference variation records that collate all studies that have characterized a specific molecular variant, and all strains that carry it. This has increased the efficiency of our storage and computation, and has also allowed us to provide more meaningful and intuitive displays for the data.

### Other nematodes

The manual curation of primary annotation for other nematode species is directed by user requests and perceived impact. Accordingly, we have begun to prioritize key parasitic species of direct relevance to human health. As a pilot project, we performed a first-pass annotation of the genome of *Brugia malayi*, a causative agent of lymphatic filariasis, manually reviewing nearly 3000 gene loci (21% of all genes) within a 6-month period. Working in collaboration with the filariasis community via FR3 (NIAID Filariasis Research Reagent Resource Center), targets for manual curation were identified based on their likely importance to the research community (e.g. putative drug targets; putative essential genes, based on *C. elegans* orthology; protein kinases; and transcription factors).

WormBase now houses the reference genomic sequence annotation for >20 nematode species. A number of these data sets originate from third-party genome sequencing and annotation projects, and WormBase’s role is primarily to add value via a number of computation analyses, display the data and make it available in standard formats. The basic workflow for integrating a genome into WormBase comprises (i) review and quality control of the primary submitted data, (ii) deployment of computational pipelines to provide additional first-pass functional annotation of the gene products and predictions of orthology and paralogy to other nematode genes and (iii) the provision of a genome browser, as well as data files in standard formats (e.g. FASTA, GFF v3) made available via our FTP site (ftp://ftp.wormbase.org). Species that we have recently brought into WormBase in this way are *Heterorhabditis bacteriophora* [a nematode used in horticulture (10)], *Bursaphelenchus xylophilus* [the pine wilt nematode (11)], *Loa loa* [a causative agent of *Loa loa* filariasis (12)], *Panagrellus redivivus* [the ‘sour paste’ nematode (13)] and *Dirofilaria immitis* [the dog heartworm (14)].

Owing to diminishing costs of sequencing, it is now becoming more common to see the initiation of multiple independent reference genome projects for a single species. This is exemplified by the cases of two particular nematode species: *Ascaris suum*, for which two independent projects have each sequenced a different tissue (15,16); and *Haemonchus contortus*, for which two independent projects have each sequenced a different key strain (17,18). To distinguish between different genome projects on our FTP site and Web site displays and services, we use the NCBI BioProject accession (http://www.ncbi.nlm.nih.gov/bioproject), which is guaranteed to be a unique handle.

## IMPROVEMENTS TO THE WEB SITE

To address increased demand for the Web site and the need to store and present growing amounts of data, we rebuilt the WormBase Web site from the ground up. Released on 30 March 2012, this rewrite included not only a brand new user interface but also new searching tools and increased user support. A new back-end architecture provides support for the site and we have begun migration of hosting to the Amazon AWS cloud.

### User interface

We designed the new user interface with the following objectives: (i) the interface should be as species-agnostic as possible, removing the emphasis on *C. elegans* when appropriate, (ii) the interface should be customizable and allow users to select which types of data they wish to see and (iii) the interface should be future-forward and permit facile changes to the content and display.

As mentioned earlier, our primary user community remains researchers using *C. elegans* as a model system. Reworking the Web site to accommodate additional species serves two purposes. First, comparative approach against closely related species is a typical use case for studying gene function and genome architecture in *C. elegans*. Second, by de-emphasizing *C. elegans*, we have made it possible to more easily support underserved research communities studying nematodes of agricultural and medical significance. To accomplish this, we added a site-wide ‘Species’ option on the main navigation bar. From here, users can toggle between species from any location on the site, see genome assembly and version information, jump directly to customized report pages and searches and so on. Precomputed homology and orthology relationships provide further means for moving quickly between species.

As the number of species and extent of data housed at WormBase continue to grow, we wanted to both create data-rich reports and also allow users to pick and choose which data are most important to them, as well as control its presentation on the page. On report pages (say, for a given gene), a navigational sidebar lists the available ‘widgets’ for that page. When a user clicks on a widget title, the corresponding widget opens. Widgets can be rearranged on the page by drag-and-drop, collapsed and dismissed as needed. A flexible single or multicolumn layout lets users build the perfect page report for the research question at hand. For users who have chosen to log in to the site, layout settings persist between sessions. Many other options for interactivity and customization have been built in to the new site. Users can log in using Google, Facebook or local WormBase credentials. Once logged in, they may save favorite pages (My Favorites) and papers (My Library).

We enhanced the ease of finding content at WormBase by building a custom search engine powered by Xapian (www.xapian.org). Users can conduct full-text searches on WormBase, and retrieve faceted results broken down by data type (e.g. gene, molecule), paper type (e.g. review, journal, article) and species. The results can be further filtered by type or species, or downloaded for further analysis.

The new interface also introduces elements created to help foster community engagement. Every page has a feedback tab prompting users to leave feedback, submit content corrections, report bugs or ask for help. Furthermore, each report page has a place for public comments, creating a low participation-barrier community annotation system.

The Perl web framework Catalyst (www.catalystframework.org) provides the core of the new site. A Model-View-Controller design implementation effectively separates concerns and allows us to create different presentations when accessing the same data. In this manner, the WormBase site can easily continue to evolve to meet user needs and expectations.

### Back-end architecture

WormBase continues to rely on AceDB as the primary platform for data integration and distribution. This single-threaded database management system is >20 years old and built before the era of multi-species whole-genome sequences and annotation. We have encapsulated the role of AceDB in the new Web site architecture by building a RESTful Application Programming Interface (API) into our application that consumes data from AceDB and supplementary MySQL databases, returning data properly structured for presentation. This encapsulation effectively decouples the Web site from the back-end store, opening the door for us to migrate to a new system in the near future.

One migration path that we have begun to explore uses the NoSQL document store CouchDB (couchdb.apache.org). In our current application, we precompute computationally intensive displays (using the RESTful API) and store the data in CouchDB as an efficient data cache. AceDB is only accessed when data do not already exist in CouchDB. We have extended this proof of concept by rewriting the Perl interface to AceDB (AcePerl) to use data loaded into a standalone CouchDB instance (Ace::Couch), completely removing the requirement for AceDB to drive the Web site.

Improvements to the WormBase Web site have not been limited to software upgrades. Most significantly, we have begun to move the entirety of the WormBase Web site to hosting on Amazon’s commercial cloud computing services. Services such as Elastic Cloud Compute (EC2) are well suited for hosting our non-sensitive information and simplify many aspects of managing the Web site. Administrative costs—both in time and money—of hosting and maintaining the Web site are greatly reduced from traditional on-site environments. Because pricing models use a ‘pay for what you use’ scheme, the costs of hosting in the cloud are comparable to or cheaper than institutional hosting when factoring in overhead costs. Moreover, additional storage and compute capacity can be added (and later removed) as needs arise without incurring capital expense. Cloud-based data are easily versioned and inexpensively archived through the use of snapshots. Finally, cloud resources can be launched in various geographical locations to provide better performance for users in different areas of the globe.

### New data visualization and mining tools

The new Web site architecture allows us to easily maintain and add new tools to the Web site. For example, popular pre-existing tools such as GBrowse and BLAST/BLAT tools were retrofitted to work with the new site structure. We have expanded the options for data mining in two significant ways. First, we have launched an instance of the InterMine [www.intermine.org; (19)] data warehousing and mining platform called WormMine. WormMine gives users new ways to query data, save and manipulate lists of objects and download data *en masse*. WormMine also increases the interoperability of WormBase with other model organism databases that have built their own InterMine instances. Second, we have opened the same RESTful API that we use to build the Web site. Developers can consume this API to create their own presentations of the WormBase data. Researchers can use this to programmatically retrieve WormBase data in a variety of formats.

### Community and user support

With the release of the new Web site, we have made it simpler for users to interact with WormBase developers and curators. A ‘Questions, Feedback & Help’ tab is visible on every page on WormBase. Submitting a query here is integrated with our mail-based help desk. User forums, Twitter, a blog and webcasts augment the direct user support that WormBase provides.

## FUTURE DIRECTIONS

The WormBase curation strategy, build process and Web site continue to evolve in response to user feedback and technical requirements. In the near future, we plan to finish relocating the Web site to the Amazon cloud. We are continuing to explore back-end replacement options for the two roles AceDB plays at WormBase: as the primary data integration platform and as a data source that drives the Web site. To accommodate increasing numbers of users accessing the WormBase Web site, we will shortly launch a version of the site optimized for mobile use to be followed by native applications for both Android and iOS.

## FUNDING

The US National Human Genome Research Institute [U41-HG002223] to WormBase and British Medical Research Council [G070119] to WormBase; P.W.S. is an investigator with the Howard Hughes Medical Institute. Funding for open access charge: US National Human Genome Research Institute [U41-HG002223].

*Conflict of interest statement*. None declared.
